# Temporal changes in serum uric acid and risk for metabolic syndrome: a longitudinal cohort study

**DOI:** 10.1186/s13098-022-00861-6

**Published:** 2022-07-06

**Authors:** Xuan Feng, Yi Guo, Huakang Tu, Shu Li, Chen Chen, Mingxi Sun, Sicong Wang, Bohan Li, Xifeng Wu, Zhenya Song

**Affiliations:** 1 Department of Big Data in Health Science School of Public Health, and Center of Clinical Big Data and Analytics of The Second Affiliated Hospital, Zhejiang University School of Medicine, Hangzhou, Zhejiang China; 2 Alibaba-Zhejiang University Joint Research Center of Future Digital Healthcare, Zhejiang, China; 3 Department of Health Management Center and Department of General Medicine, The Second Affiliated Hospital Zhejiang University School of Medicine, Hangzhou, Zhejiang China; 4 National Institute for Data Science in Health and Medicine, Zhejiang University, Hangzhou, Zhejiang China; 5 Cancer Center, Zhejiang University, Hangzhou, Zhejiang China; 6 School of Medicine and Health Science, George Washington University, Washington, DC USA; 7 The Key Laboratory of Intelligent Preventive Medicine of Zhejiang Province, Hangzhou, 310058 Zhejiang China

**Keywords:** Metabolic syndrome, Serum uric acid, Longitudinal cohort, Joint effect

## Abstract

**Background:**

Studies suggested elevated serum uric acid (SUA) levels are associated with metabolic syndrome (MetS). However, it remains unclear whether baseline SUA and temporal changes predict MetS. The study aimed to investigate the association of baseline SUA and its temporal longitudinal changes with subsequent risk of MetS.

**Methods:**

We conducted a retrospective longitudinal cohort study among 44,176 healthy participants aged 18 years and older without MetS at enrollment. The baseline levels and longitudinal changes of SUA were categorized by gender-specific quintiles. Participants were followed to identify newly developed MetS. We employed Cox model to investigate the relationship between SUA and MetS in men and women separately.

**Results:**

During a median follow-up of 2.4 years, 5461 (12.36%) participants developed MetS. After adjustment of demographic, major clinical factors, a higher level of baseline SUA was associated with a significant higher risk of MetS. The corresponding HRs (95% CIs) comparing participants at extreme quintiles were 2.59 (2.32, 2.88) in men and 2.87 (2.41, 3.43) in women. Larger longitudinal absolute increase in SUA was also related to an increases risk of MetS (top vs bottom quintile, 1.70 [1.53, 1.89] in men and 1.94 [1.65, 2.28] in women), regardless the level of baseline SUA. Similarly, the HRs about SUA longitudinal percentage changes were 1.74 (1.56, 1.94) in men and 2.01 (1.69, 2.39) in women, respectively. Moreover, we observed the highest risk of MetS among participants with both higher baseline SUA and larger longitudinal increase in SUA.

**Conclusion:**

Higher baseline SUA and larger temporal increase in SUA independently predicted risk of MetS, highlighting the importance of longitudinal SUA monitoring and management for primary prevention of MetS in the general population.

## Background

Metabolic syndrome (MetS), a clinical syndrome, is a complex metabolic disorder in sugar, protein, and lipid metabilsm [1]. MetS has gradually become a global epidemic and a serious public problem affecting human health. According to the National Health and Nutrition Examination Survey (NHANES) survey data, the overall crude prevalence of MetS in the USA increased from 32.5% to 36.9% from 2011–2012 to 2015–2016 [2]. The Asia Pacific region is also facing a serious epidemic with a rapidly increasing incidence of MetS [3]. In China, the overall age-standardized prevalence is about 20% in 2009 [4]. A 2018 review reported that nearly over one billion people across the globe now suffered from MetS [5]. In addition, a large body of literature has demonstrated that MetS increases the risk of type 2 diabetes, cancer, cardiovascular disease and all-cause mortality [6–10].

Serum uric acid (SUA) is the end product of purine metabolism or purine nucleotide catabolism [11]. When the regulation of SUA production and excretion is out of balance, the levels of SUA become abnormal. It has been strongly demonstrated that elevated SUA level is closely related to diabetes, hypertension, obesity, renal function decline, and cardiovascular disease, most of which are principal contributors in the development and progression of MetS [12–17]. At present, hyperuricemia has not been included in the diagnostic criteria of MetS; however, some researches findings illustrated that the SUA level was highly correlated with the risk of MetS in different populations [18–20]. A meta-analysis of 11 cohort studies suggested that the combined RR of MetS risk was 1.72 (1.45, 2.03) comparing the top SUA level category to the lowest SUA level category, and dose–response analysis indicated that the risk of developing MetS increased 1.30 (1.22, 1.38) times for per 1 mg/dL SUA increment [21]. Even the relationship between higher SUA levels and increased MetS were widely reported, most previous studies were conducted using cross-sectional or cohort designs, with SUA levels measured once at baseline. It is still unclear whether the temporal dynamic change of SUA is an independent risk factor for MetS, especially for those with baseline SUA levels within the normal reference range.

The study aimed to assess the associations of baseline and temporal changes in SUA with the risk of developing MetS and their joint effects on MetS risk in a Chinese general population using longitudinal physical examination data.

## Methods

### Participants

The study design was a retrospective longitudinal cohort study. The data of participants originated from physical examination databases of desensitization in the Second Affiliated Hospital of Zhejiang University School of Medicine. The baseline data were collected from people who first underwent physical examinations from 1 January 2013 to 31 December 2015, with follow-up visits approximately once a year, and participants were followed up to 31 December 2019.

The inclusion and exclusion criteria of the study participants were as follows:

Inclusion criteria: age ≥ 18 years old; undiagnosed with MetS at baseline; complete information of all diagnostic indicators of MetS;

Exclusion criteria: no follow-up visits; history of diabetes, hypertension, coronary heart disease, stroke and other cardiovascular and cerebrovascular diseases, malignant tumors, severe liver and kidney diseases;

In brief, 157,993 people participated in the medical examination from 2013 to 2015. After excluding 6857 people with incomplete diagnosis information of MetS, 31,927 people with MetS, 13,602 people with previous major chronic diseases, 77 people under the age of 18, and 61,352 people with no follow-up visits, finally, 44,176 people were included and retained as our research participants for later analysis (21,972 men and 22,204 women).

### Data collection and variable definition

General demographic characteristics (sex, age), physical examination information (height, weight, blood pressure), smoking status (yes/no), drinking (yes/no), laboratory examination information (fasting blood) of the participants and the date of health checkups were all collected from the database. In detail, sex, age, smoking, drinking, body mass index [BMI, weight/height^2^ (kg/m^2^)], fasting blood glucose (FBG, mmol/L), systolic blood pressure (SBP, mmHg),diastolic blood pressure (DBP, mmHg), triglycerides (TG, mmol/L), high-density lipoprotein cholesterol (HDL-C, mmol/L), low-density lipoprotein cholesterol (LDL-C, mmol/L), total cholesterol (TC, mmol/L), serum uric acid (SUA, μmol/L), serum creatinine (SCr, μmol/L) were collected from 2013 to 2019. If the subject had an outcome before the end of the study period, no further data would be collected.

### Definitions of exposure and outcome

The exposures were baseline SUA, longitudinal absolute and percentage changes of SUA. We defined the last SUA levels as the last follow-up SUA value. The longitudinal absolute change in SUA was obtained: the last follow-up SUA—the baseline SUA. The percent change of SUA was calculated as follows: (absolute change in SUA/the baseline SUA) × 100%. Furthermore, hyperuricemia was defined as SUA ≥ 420 μmol/L in men and SUA ≥ 360 μmol/L in women [22].

The outcome was new-onset MetS in follow-up period. Diagnosis of MetS was based on a joint statement of the International Diabetes Federation (IDF), American Heart Association/National Heart, Lung and Blood Institute (AHA/NHLBI), World Heart Federation (WHF), International Atherosclerosis Society (IAS) and International Association for the Study of Obesity (IASO) [23], and MetS was defined as having any three or more of the following five components:

- Elevated blood pressure: systolic blood pressure (SBP)/diastolic blood pressure (DBP) ≥ 130/85 mmHg, or taking anti-hypertensive medication for hypertension;
- Higher triglycerides: TG ≥ 1.7 mmol/L (150 mg/dL), or having been treated for this dyslipidemia;
- Low high-density lipoprotein cholesterol: HDL-C < 1.0 mmol/L (39 mg/dL) in men and HDL-C < 1.30 mmol/L (50 mg/dL) in women, or drug treatment for lipid abnormality;
- High fasting blood glucose: FBG ≥ 5.6 mmol/L (100 mg/dL), or having received hypoglycemic therapy or insulin therapy;
- Abdominal obesity (waist circumference, WC): WC ≥ 90 cm in men, WC ≥ 80 cm in women;

Because the information of WC was lacking, we took BMI as an alternative indicator of obesity, and BMI ≥ 25 kg/m^2^ was considered as a boundary according to the Chinese Medical Association Diabetes Association report in 2004 [24]. BMI was significantly strongly correlated with WC in MetS patients [25].

### Statistical analysis

Statistical analysis was performed by R software (version 4.00). Continuous variables that didn’t fit a normal distribution were represented by the median and interquartile range [M (P_25_, P_75_)] and Kruskal–Wallis test was employed to compare the differences between the groups. Categorical variables were expressed as percentages (%) and were analyzed using Chi-square (χ^2^) tests in groups.

Cox model was used to estimate hazard ratios (HRs) and corresponding 95% confidence intervals (CIs) of the relationship between the SUA and MetS. In the Model 1, we adjusted for baseline age (continuous), smoking (yes /no), and drinking (yes/no). In the Model 2, we adjusted for TC (continuous), LDL-C (continuous) and SCr (continuous) in addition to covariates in the Model 1. Meanwhile, to assess the relationship of longitudinal changes in SUA with incident MetS, the baseline SUA (continuous) was additionally adjusted for in Model 1 and 2. The combined effects of baseline and changes of SUA were evaluated using Cox model adjusted for baseline age, smoking, drinking, TC, LDL-C, and SCr. We divided the baseline SUA into three groups based on the thresholds for diagnosis of hyperuricemia (men: 420 μmol/L, women: 360 μmol/L) and their approximate median baseline SUA (men: 360 μmol/L, women: 260 μmol/L) by gender. We defined three groups as low, moderate and high baseline group. Then, we divided longitudinal changes of SUA into five groups based on their quintiles (Q1, Q2, Q3, Q4, Q5). In this way, we can conduct the analyses of the joint effect of baseline and longitudinal changes of SUA for MetS incidence through the generated 15 groups.

All statistical tests were two-sided, and *P* < 0.05 was considered as statistical significant.

## Results

### Baseline characteristics of participants

There were 44,176 participants in the study, and 21,972 men and 22,204 women were included and followed up. The baseline age ranged from 18 to 92 years, with a median baseline age of 37 years. The median follow-up time was 2.4 (1.1, 4.0) years. During the follow up period, 5461 participants developed MetS, among which 3842 were men and 1619 were women. The incidence rate of MetS was 12.36% in general and it was significantly higher in men (3842 cases, 17.49% incidence) than in women (1619 cases, 7.29%). The overall incidence density of MetS was 4.15/100 person-years (men: 5.92/100 person-years, women: 2.43/100 person-years).

The baseline characteristics of participants according to quintiles of baseline SUA levels were presented in Table 1. The value of SBP, DBP, TG, LDL, TG, BMI, FBG and SCr in the top quintile of baseline SUA group were significantly higher than that in the lowest quintile group (*P* < 0.001) and HDL-C was lower in the top quintile group compared with the lowest quintile group in all participants (*P* < 0.001).

**Table 1:** The baseline characteristics of participants by different levels of baseline serum uric acid

	All (n = 44,176)	Quintile of baseline serum uric acid					*P*
		Q1 (n = 8655)	Q2 (n = 8831)	Q3 (n = 8954)	Q4 (n = 8812)	Q5 (n = 8924)	
Age (year)	37 (30, 47)	36 (29, 44)	37 (29, 46)	37 (30, 48)	38 (30, 48)	38 (31, 48)	< 0.001
Gender							< 0.001
Men	21,972 (49.7%)	488 (2.2%)	1579 (7.2%)	4450 (20.2%)	7006 (31.9%)	8449 (38.5%)	
Women	22,204 (50.3%)	8167 (36.8%)	7252 (32.7%)	4504 (20.3%)	1806 (8.1%)	475 (2.1%)	
Smoking							< 0.001
No	36,643 (82.9%)	8465 (23.1%)	8272 (22.6%)	7470 (20.4%)	6442 (17.6%)	5994 (16.3%)	
Yes	7533 (17.1%)	190 (2.5%)	559 (7.4%)	1484 (19.7%)	2370 (31.5%)	2930 (38.9%)	
Drinking							< 0.001
No	31,114 (70.4%)	7983 (25.6%)	7638 (24.5%)	6449 (20.7%)	4966 (16.1%)	4078 (13.1%)	
Yes	13,062 (29.6%)	672 (5.1%)	1193 (9.1%)	2505 (19.2%)	3846 (29.4%)	4846 (37.2%)	
SBP (mmHg)	116 (107, 124)	111 (103, 119)	112 (104, 121)	116 (107, 124)	118 (110, 127)	119 (113, 128)	< 0.001
DBP (mmHg)	70 (63, 77)	66 (61, 73)	67 (61, 74)	70 (63, 77)	72 (66, 79)	73 (67, 80)	< 0.001
BMI (kg/m^2^)	22.1 (20.3, 24.0)	20.7 (19.3, 22.2)	21.2 (19.6, 22.9)	22 (20.3, 23.7)	22.9 (21.1, 24.5)	23.8 (22.1, 25.2)	< 0.001
TG (mmol/L)	1.04 (0.76, 1.44)	0.82 (0.64, 1.09)	0.89 (0.69, 1.19)	1.04 (0.78, 1.41)	1.17 (0.87, 1.57)	1.37 (1.02, 1.86)	< 0.001
HDL-C (mmol/L)	1.39 (1.19, 1.62)	1.55 (1.35, 1.77)	1.5 (1.30, 1.73)	1.39 (1.20, 1.61)	1.3 (1.13, 1.50)	1.22 (1.08, 1.41)	< 0.001
LDL-C (mmol/L)	2.65 (2.24, 3.15)	2.43 (2.04, 2.89)	2.56 (2.14, 3.01)	2.67 (2.26, 3.15)	2.78 (2.37, 3.26)	2.87 (2.48, 3.36)	< 0.001
TC (mmol/L)	4.73 (4.20, 5.31)	4.56 (4.05, 5.14)	4.67 (4.14, 5.23)	4.74 (4.22, 5.31)	4.81 (4.27, 5.36)	4.86 (4.35, 5.47)	< 0.001
FBG (mmol/L)	4.94 (4.68, 5.23)	4.93 (4.68, 5.19)	4.92 (4.67, 5.20)	4.94 (4.67, 5.23)	4.97 (4.70, 5.26)	4.96 (4.69, 5.24)	< 0.001
SUA (μmol/L)	308 (255, 368)	219 (200, 232)	265 (254, 275)	307 (296, 317)	353 (341, 367)	420 (398, 450)	< 0.001
SCr (μmol/L)	63 (53, 75)	52 (47, 57)	55 (50, 62)	63 (55, 73)	72 (63, 79)	76 (69, 82)	< 0.001

Data are presented as median (interquartile range) values for continuous variables, percentage form (%) for categorical variables

*MetS* metabolic syndrome, *DBP* diastolic blood pressure, *SBP* systolic blood pressure, *BMI* body mass index, *TG* triglycerides, *HDL-C* high-density lipoprotein cholesterol, *LDL-C* low-density lipoprotein cholesterol, *TC* total cholesterol, *FBG* fasting blood glucose, *SUA* serum uric acid, *SCr* serum creatinine

*P* values were calculated using analysis of Kruskal–Wallis test and Chi-square test for continuous and categorical variables, respectively

### Association between baseline SUA and incident MetS

As shown in Table 2, the risk of MetS was increased for the higher levels of baseline SUA and there was a dose–response manner in SUA levels and risk of developing MetS in both men and women (*P* for trend < 0.001). In the unadjusted Model, the risks of developing MetS were increased in the higher quintiles compared with the lowest quintile of SUA, with HRs (95% CI) of 1.38 (1.22, 1.55) for quintile 2, 1.57 (1.40, 1.76) for quintile 3, 1.97 (1.76, 2.20) for quintile 4, and 2.73 (2.45, 3.04) for quintile 5 in men respectively, and the HR were 1.19 (0.97, 1.47), 1.59 (1.31, 1.93), 2.43 (2.03, 2.91), 3.55 (2.99, 4.22) in women. In the Model 2, we also found that a significant increase in the risk of suffering from MetS for participants with baseline SUA levels in the second quintile or more as compared with the bottom quintile (*P* < 0.001). In detail, compared with the lowest quintile, the HR (95% CI) for the second, the third, the fourth and the top quintile of baseline SUA values were 1.38 (1.23, 1.56), 1.53 (1.36, 1.72), 1.91 (1.70, 2.14), 2.59 (2.32, 2.88), respectively and *P* for trend < 0.001 in men. There were also respectively a 1.16 (0.94, 1.43), 1.51 (1.25, 1.84), 2.17 (1.81, 2.61), 2.87 (2.41, 3.43) fold increased risk of developing MetS in participants across other four higher quintiles of baseline SUA levels in women. When SUA was analyzed as a continuous variable in model 2, per additional 10 units increase of SUA was associated with a 5% (5%, 6%) higher MetS risk in men, 8% (7%, 9%) higher in women.

**Table 2:** Association between baseline SUA levels and incident MetS by gender

Categories	Event/total	Unadjusted Model HR (95%CI) *P* Value		Model 1 HR (95%CI) *P* Value		Model 2 HR (95%CI) *P* Value	
Baseline SUA in men							
Q1 (< 313 μmol/L)	475/4380	1 (reference)		1 (reference)		1 (reference)	
Q2 (313 to < 346 μmol/L)	618/4290	1.38 (1.22, 1.55)	< 0.001	1.41 (1.25, 1.59)	< 0.001	1.38 (1.23, 1.56)	< 0.001
Q3 (346 to < 379 μmol/L)	706/4437	1.57 (1.40, 1.76)	< 0.001	1.60 (1.43, 1.80)	< 0.001	1.53 (1.36, 1.72)	< 0.001
Q4 (379 to < 419 μmol/L)	883/4417	1.97 (1.76, 2.20)	< 0.001	2.01 (1.80, 2.25)	< 0.001	1.91 (1.70, 2.14)	< 0.001
Q5 (≥ 419 μmol/L)	1160/4448	2.73 (2.45, 3.04)	< 0.001	2.77 (2.49, 3.08)	< 0.001	2.59 (2.32, 2.88)	< 0.001
* P* for trend			< 0.001		< 0.001		< 0.001
Per 10 units increase	3842/21972	1.06 (1.05, 1.06)	< 0.001	1.06 (1.05, 1.06)	< 0.001	1.05 (1.05, 1.06)	< 0.001
Baseline SUA in women							
Q1 (< 221 μmol/L)	166/4415	1 (reference)		1 (reference)		1 (reference)	
Q2 (221 to < 247 μmol/L)	200/4313	1.19 (0.97, 1.47)	0.094	1.19 (0.96, 1.46)	0.105	1.16 (0.94, 1.43)	0.155
Q3 (247 to < 272 μmol/L)	273/4484	1.59 (1.31, 1.93)	< 0.001	1.53 (1.26, 1.86)	< 0.001	1.51 (1.25, 1.84)	< 0.001
Q4 (272 to < 304 μmol/L)	404/4520	2.43 (2.03, 2.91)	< 0.001	2.25 (1.88, 2.70)	< 0.001	2.17 (1.81, 2.61)	< 0.001
Q5 (≥ 304 μmol/L)	576/4472	3.55 (2.99, 4.22)	< 0.001	2.95 (2.48, 3.52)	< 0.001	2.87 (2.41, 3.43)	< 0.001
* P* for trend			< 0.001		< 0.001		< 0.001
Per 10 units increase	1619/22204	1.09 (1.08, 1.10)	< 0.001	1.08 (1.07, 1.09)	< 0.001	1.08 (1.07, 1.09)	< 0.001

*SUA* serum uric acid, *MetS* metabolic syndrome, *CI* confidence interval, *TC* total cholesterol, *LDL-C* low-density lipoprotein cholesterol, *SCr* serum creatinine

Model 1: adjusted for baseline age, smoking status, drinking status

Model 2: adjusted for baseline TC, LDL-C and SCr, in addition to covariates in Model 1

### Association between longitudinal changes in SUA and incident MetS

In the Table 3, the relationship between the longitudinal change of SUA and the occurrence of MetS was demonstrated. Compared to the lowest quintile of absolute changes of SUA, the adjusted HRs (95% CI) for developing MetS in other four quintiles were 1.14 (1.02, 1.26), 1.38 (1.24, 1.53), 1.45 (1.30, 1.61) and 1.70 (1.53, 1.89), respectively in men, and the corresponding HRs in women were 1.40 (1.18, 1.66), 1.47 (1.23, 1.75), 1.63 (1.37, 1.94) and 1.94 (1.65, 2.28). Similar but more significant results were observed with respect to the percentage changes of SUA. A trend for an association was observed for the percentage changes of SUA and risk of MetS (the HR per 10% increase, 1.16 [1.13, 1.19] in men, 1.15 [1.11, 1.18] in women). When the percentage change in SUA was assessed as quintiles, the HRs across the quintiles were 1.00, 1.18 (1.06, 1.31), 1.41 (1.27, 1.56), 1.50 (1.35, 1.66) and 1.74 (1.56, 1.94) in men, respectively and HRs were 1.00, 1.44 (1.22, 1.71), 1.49 (1.26, 1.77), 1.70 (1.43, 2.01) and 2.01 (1.69, 2.39) in women. There was a significant increase in the risk of MetS incidence across the quintiles of the percentage changes in SUA (*P*_trend_ < 0.001).

**Table 3:** Association between SUA changes and incident MetS by gender

Categories	Event/total	Unadjusted model HR (95%CI) *P* Value		Model 1 HR (95%CI) *P* Value		Model 2 HR (95%CI) *P* Value	
Men							
Longitudinal absolute changes of SUA							
Q1 (< − 32 μmol/L)	742/4289	1 (reference)		1 (reference)		1 (reference)	
Q2 (− 32 to < − 6 μmol/L)	711/4493	0.90 (0.81, 1.00)	0.046	1.14 (1.03, 1.27)	0.012	1.14 (1.02, 1.26)	0.016
Q3 (− 6 to < 15 μmol/L)	723/4234	0.99 (0.89, 1.10)	0.876	1.38 (1.24, 1.53)	< 0.001	1.38 (1.24, 1.53)	< 0.001
Q4 (15 to < 42 μmol/L)	793/4557	0.99 (0.90, 1.10)	0.878	1.46 (1.31, 1.62)	< 0.001	1.45 (1.30, 1.61)	< 0.001
Q5 (≥ 42 μmol/L)	873/4399	1.10 (1.00, 1.22)	0.050	1.72 (1.55, 1.91)	< 0.001	1.70 (1.53, 1.89)	< 0.001
* P* for trend			0.007		< 0.001		< 0.001
Per 10 units increase	3842/21972	1.01 (1.00, 1.01)	0.022	1.04 (1.03, 1.05)	< 0.001	1.04 (1.03, 1.05)	< 0.001
Longitudinal percent changes of SUA							
Q1 (< − 8.39%)	704/4393	1 (reference)		1 (reference)		1 (reference)	
Q2 (− 8.39% to < − 1.77%)	738/4399	1.03 (0.93, 1.15)	0.543	1.18 (1.07, 1.31)	0.002	1.18 (1.06, 1.31)	0.002
Q3 (− 1.77% to < 4.37%)	788/4394	1.14 (1.03, 1.26)	0.014	1.41 (1.27, 1.56)	< 0.001	1.41 (1.27, 1.56)	< 0.001
Q4 (4.37% to < 12.14%)	800/4388	1.12 (1.02, 1.24)	0.023	1.51 (1.36, 1.68)	< 0.001	1.50 (1.35, 1.66)	< 0.001
Q5 (≥ 12.14%)	812/4398	1.10 (1.00, 1.22)	0.062	1.76 (1.58, 1.96)	< 0.001	1.74 (1.56, 1.94)	< 0.001
* P* for trend			0.018		< 0.001	< 0.001	
Per 10% increase	3842/21972	1.03 (1.00, 1.05)	0.027	1.16 (1.13, 1.19)	< 0.001	1.16 (1.13, 1.19)	< 0.001
Women							
Longitudinal absolute changes of SUA							
Q1 (< − 28 μmol/L)	264/4328	1 (reference)		1 (reference)		1 (reference)	
Q2 (− 28 to < − 5 μmol/L)	290/4422	1.05 (0.89, 1.25)	0.541	1.38 (1.17, 1.64)	< 0.001	1.40 (1.18, 1.66)	< 0.001
Q3 (− 5 to < 14 μmol/L)	285/4360	1.03 (0.87, 1.21)	0.772	1.47 (1.23, 1.75)	< 0.001	1.47 (1.23, 1.75)	< 0.001
Q4 (14 to < 37 μmol/L)	338/4516	1.14 (0.97, 1.33)	0.120	1.68 (1.41, 1.99)	< 0.001	1.63 (1.37, 1.94)	< 0.001
Q5 (≥ 37 μmol/L)	442/4578	1.36 (1.17, 1.58)	< 0.001	2.03 (1.73, 2.39)	< 0.001	1.94 (1.65, 2.28)	< 0.001
* P* for trend			< 0.001		< 0.001		< 0.001
Per 10 units increase	1619/22204	1.03 (1.02, 1.04)	< 0.001	1.05 (1.04, 1.07)	< 0.001	1.05 (1.04, 1.06)	0.011
Longitudinal percent changes of SUA							
Q1 (< − 10.19%)	242/4441	1 (reference)		1 (reference)		1 (reference)	
Q2 (− 10.19 to < − 1.90%)	316/4442	1.29 (1.09, 1.52)	0.003	1.48 (1.25, 1.76)	< 0.001	1.44 (1.22, 1.71)	< 0.001
Q3 (− 1.90% to < 5.75%)	319/4443	1.26 (1.06, 1.49)	0.007	1.50 (1.26, 1.78)	< 0.001	1.49 (1.26, 1.77)	< 0.001
Q4 (5.75% to < 15.29%)	358/4435	1.38 (1.17, 1.62)	< 0.001	1.79 (1.51, 2.13)	< 0.001	1.70 (1.43, 2.01)	< 0.001
Q5 (≥ 15.29%)	384/4443	1.37 (1.16, 1.61)	< 0.001	2.13 (1.79, 2.53)	< 0.001	2.01 (1.69, 2.39)	< 0.001
* P* for trend			< 0.001		< 0.001		< 0.001
Per 10% increase	1619/22204	1.05 (1.02, 1.08)	< 0.001	1.16 (1.12, 1.19)	< 0.001	1.15 (1.11, 1.18)	< 0.001

*SUA* serum uric acid, *MetS* metabolic syndrome, *CI* confidence interval, *TC* total cholesterol, *LDL-C* low-density lipoprotein cholesterol, *SCr* serum creatinine

Model 1: adjusted for baseline age, smoking status, drinking status, SUA

Model 2: adjusted for baseline TC, LDL-C, and SCr, in addition to covariates in Model 1

### Joint associations of baseline SUA and longitudinal changes in SUA with incident MetS

**Fig. 1:**
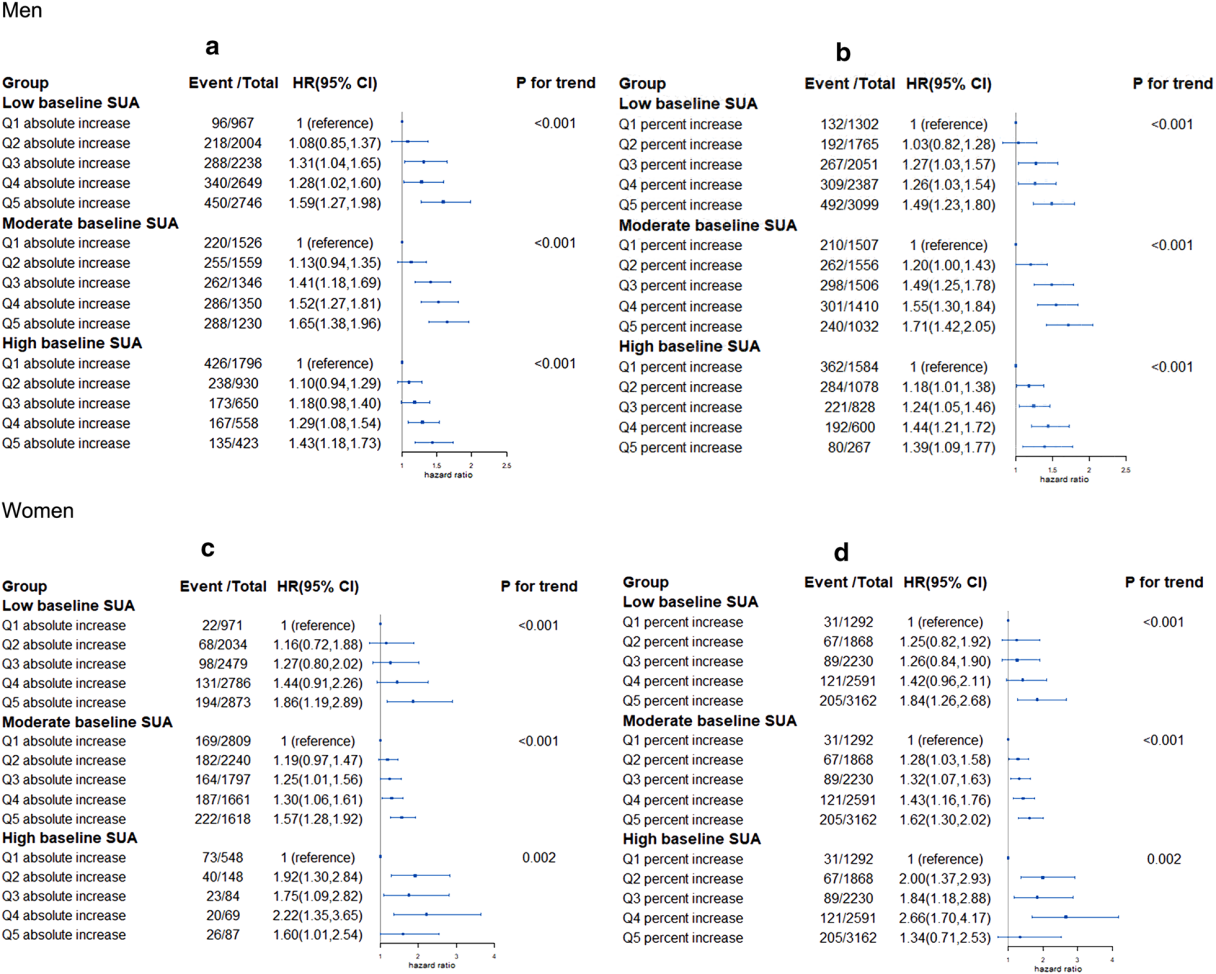
Associations of the longitudinal changes of SUA with incident MetS by different baseline SUA levels. **a** SUA longitudinal absolute changes in men. **b** SUA longitudinal percent changes in men. **c** SUA longitudinal absolute changes in women. **d** SUA longitudinal percent changes in women. *HR* hazard ratio, *CI* confidence interval. Baseline SUA group: men (< − 360 μmol/L, 360 to < 420 μmol/L, ≥ 420 μmol/L); women (< − 260 μmol/L, 260 to < 360 μmol/L, ≥ 360 μmol/L). Cut-off values of quintiles of SUA absolute changes: men (< − 32 μmol/L, − 32 to < − 6 μmol/L, − 6 to < 15 μmol/L, 15 to < 42 μmol/L, ≥ 42 μmol/L); women (< − 28 μmol/L, − 28 to < − 5 μmol/L, − 5 to < 14 μmol/L, 14 to < 37 μmol/L, ≥ 37 μmol/L). Cut-off values of quintiles of SUA percent changes: men (< − 8.39%, − 8.39% to < − 1.77%, − 1.77% to < 4.36%, 4.36% to < 12.14%, ≥ 12.14%); women (< − 10.19%, − 10.19% to < − 1.90%, − 1.90% to < 5.75%, 5.75% to < 15.29%, ≥ 15.29%). The analysis adjusted for baseline age (continuous, years), smoking status (yes and no), drinking status (yes and no), TC (mmol/L), LDL-C (mmol/L) and SCr (μmol/L)

Figure 1 presents the longitudinal increases in SUA with MetS incidence by different SUA levels at baseline. We found that the risk of MetS was significantly increased from quintile 1 to quintile 5 of longitudinal augments in SUA, and this trend was particularly pronounced in low and moderate baseline SUA groups. In the moderate baseline SUA group, the HR of MetS incidence in Q5 absolute increase group was 1.65 (1.38, 1.96) compared with participants in Q1 absolute increase group (Fig. 1.a) in men, and 1.57 (1.28, 1.92) in women (Fig. 1c). Fig. 1b, d visually showed that the overall risk of incident MetS increased as increases in longitudinal percentage of SUA. The participants of the top quintile of longitudinal percentage increase in SUA had a 71% higher risk of MetS compared with those of the first group in moderate baseline SUA group in men (Fig. 1a), 62% higher in women (Fig. 1c).

**Fig. 2:**
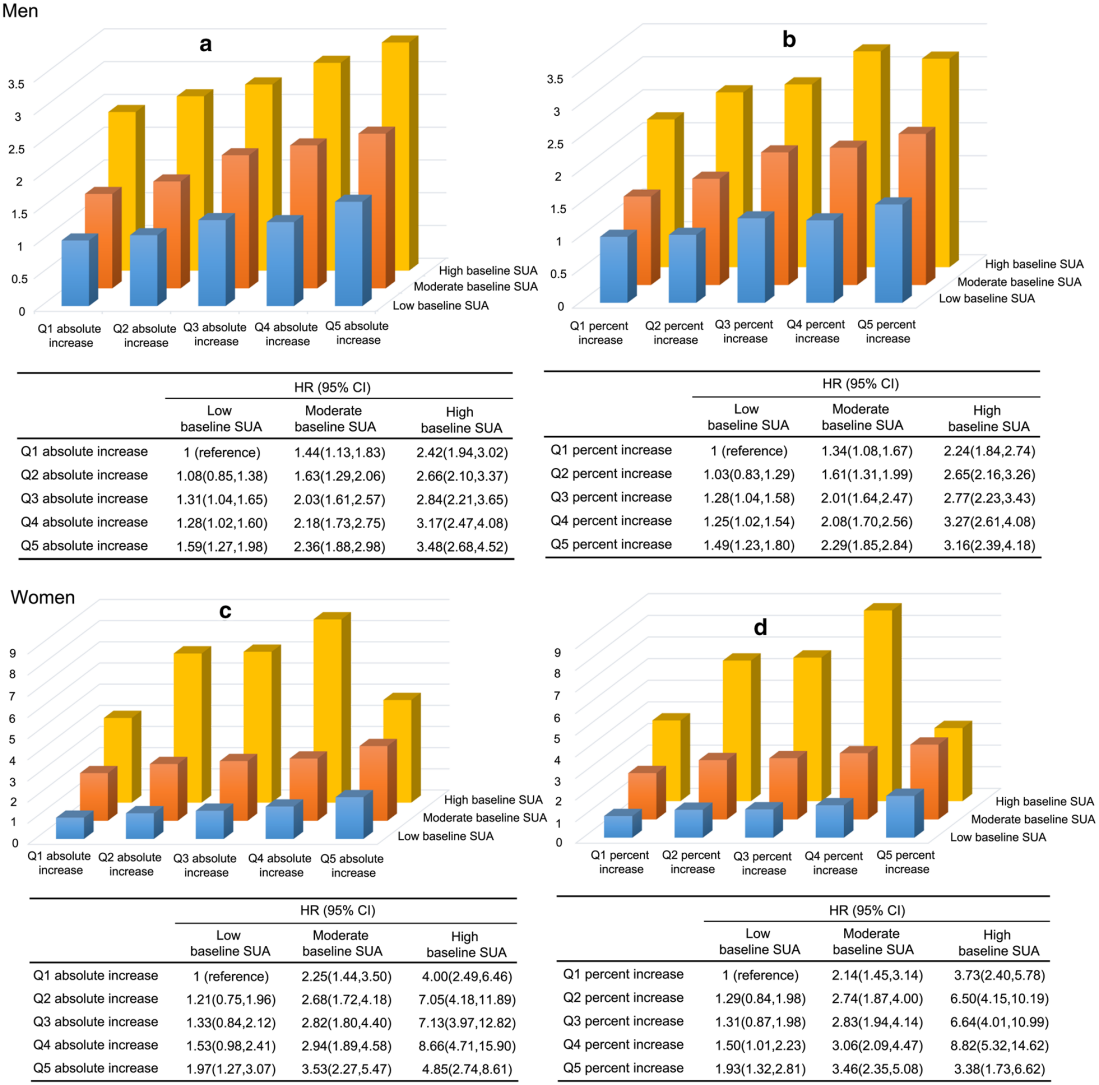
Joint associations of baseline SUA levels and longitudinal changes in SUA levels with incident MetS. **a** SUA longitudinal absolute changes in men. **b** SUA longitudinal percent changes in men. **c** SUA longitudinal absolute changes in women. **d** SUA longitudinal percent changes in women. *HR* hazard ratio, *CI* confidence interval. Baseline SUA group: men (< − 360 μmol/L, 360 to < 420 μmol/L, ≥ 420 μmol/L); women (< − 260 μmol/L, 260 to < 360 μmol/L, ≥ 360 μmol/L). Cut-off values of quintiles of SUA absolute changes: men (< − 32 μmol/L, − 32 to < − 6 μmol/L, − 6 to < 15 μmol/L, 15 to < 42 μmol/L,  ≥ 42 μmol/L); women (< − 28 μmol/L, − 28 to < − 5 μmol/L, − 5 to < 14 μmol/L, 14 to < 37 μmol/L,  ≥ 37 μmol/L). Cut-off values of quintiles of SUA percent changes: men (< − 8.39%, − 8.39% to < − 1.77%, − 1.77% to < 4.36%, 4.36% to < 12.14%,  ≥ 12.14%); women (< − 10.19%, − 10.19% to < − 1.90%, − 1.90% to < 5.75%, 5.75% to < 15.29%,  ≥ 15.29%). The analysis adjusted for baseline age (continuous, years), smoking status (yes and no), drinking status (yes and no), TC (mmol/L), LDL-C (mmol/L) and SCr (μmol/L)

Figure 2 showed that an increased risk of MetS with increased baseline SUA and increases in longitudinal SUA changes in both men and women. Compared to the group with low baseline SUA and Q1 absolute longitudinal increase, the risks in the other 14 groups increased 1.08–3.48 folds in men and 1.21–8.66 folds in women, with the highest risk observed in the group with high baseline SUA and Q5 absolute longitudinal increase in men and in the group with high baseline SUA and Q4 absolute longitudinal increase in women (Fig. 2a and b). Similar results were observed for the joint associations between baseline SUA and longitudinal percent changes (Fig. 2b and d). There was a linear trend, dose–response fashion in the hazard of MetS across the 15 categories in both men and women (*P*_trend_ < 0.001), although there was a fluctuation of HR in high baseline SUA group in women.

## Discussion

To our knowledge, the study was the first study to assess the associations of temporal changes of SUA with MetS in a large Chinese general population by gender. In this study, we assessed that both baseline SUA and longitudinal changes in SUA were significantly associated with the risk of MetS incidence. In addition, we also found that longitudinal increases in SUA could increase the risk of incident MetS, even when baseline SUA was within the normal reference range.

The relationship between baseline SUA and MetS has been extensively studied previously. Nurshad Ali found that elevated baseline SUA was significantly positively associated with MetS and its components in Bangladeshi general adults [26]. A meta-analysis study suggested that the pooled HR for MetS incidence was 1.55 (1.40, 1.70) on the comparison between the top and the bottom category, and the risk of MetS increased 5% for per 1 mg/dL increase [27]. A nation-wide cohort study in China demonstrated that there was a 55% increase in the risk of MetS in the highest group compared to the lowest baseline SUA group, and the relative risk was 1.32 (1.25, 1.40) for the top versus the bottom SUA category in a meta-analysis [28]. Our study obtained similar results, although the effect estimates were slightly different. This suggests that elevated SUA level  increases the risk of developing MetS. However, the underlying biological mechanism of SUA triggering MetS remains unclear. Some other studies found that high SUA can activate the chronic inflammatory response, oxidative stress, induce endothelial dysfunction and activate the renin angiotensin aldosterone system to further aggravate the degree of insulin resistance [29–33].

The study also found that the longitudinal increase of SUA was positively linked with the MetS incidence, independent of baseline SUA. What we observed were roughly in line with the results of baseline SUA. The similarity is that in a population-based study of 6083 Norwegian adults, the risk of MetS incidence was increased 1.28 (1.16, 1.42) fold for per 59 μmol/L increase of longitudinal SUA among all populations [34]. Another retrospective cohort study among 407 Japanese community-dwelling women manifested that the risk of MetS increased 2.49 (1.38, 4.47) fold comparing the third tertile to the first tertile of longitudinal increase in SUA [35]. Our study added to the credibility of the results of previous epidemiological studies in different populations and our findings also further demonstrated that the strengths of associations were different among men and women.

We also focused on the effect of SUA changes patterns on MetS incidence, and found that the risk of MetS increased as SUA increased longitudinally, even when baseline SUA levels were in the normal reference range. This relationship has rarely been reported in previous studies. We found that the risk of MetS in participants with low or moderate baseline SUA but high longitudinal increase was roughly the same as that in participants with high baseline SUA and minimal longitudinal increase. This meant that those with normal baseline SUA levels but high longitudinal increase had a higher risk of developing MetS. Previous studies showed that high SUA was positively correlated with MetS incidence [36, 37], but it was not enough to just focus on baseline levels. Longitudinal increases of SUA were also associated with higher risk of MetS. Therefore, we should dynamically monitor the SUA changes.

Our findings have some implications. Firstly, important guiding significance for health management of patients with hyperuricemia. Secondly, the vital importance of lifelong SUA control. Thirdly, early identification of high risk group of MetS. There could be key opportunities for intervention in high SUA adults because certain factors, such as healthy lifestyles, eating fruits, are important to improve the outcomes of hyperuricemia and to prevent MetS [38–40]. In addition, clinicians and primary health workers should be made aware of the important significance of long-term SUA surveillance, and the optimal SUA value should be investigated in future studies. Therefore, dynamic monitoring of SUA is essential in routine clinical practice.

The main advantages of this study are cohort study design, large sample sizes and comprehensively exploring the relationship between temporary changes of SUA and MetS. However, some limitations should be considered. First, the study data was from only one hospital which may cause selection bias. Second, we used BMI to replace WC to indicate abdominal obesity, which may lead to bias in the diagnosis of MetS, but it has been reported that BMI can replace WC to some extent in MetS populations. Third, we don't take dietary habits or other lifestyle behaviors into consideration due to not having sufficient information. Large-scale prospective cohort studies are necessary to verify the relationship between SUA temporary changes and MetS. Also, additional studies should be undertaken to clarify the underlying molecular mechanism linking SUA to MetS.

## Conclusion

Our study showed that the temporary increases of SUA were positively related to MetS in general population, even among those whose baseline SUA were in the normal reference range. Those with high baseline SUA and with greater longitudinal temporal increase were at greatest risk of developing MetS.

## Data Availability

The datasets generated and/or analyzed during the current study are not publicly available but are available from the corresponding author on reasonable request.

## Abbreviations

MetS: Metabolic syndrome
DBP: Diastolic blood pressure
SBP: Systolic blood pressure
BMI: Body mass index
TG: Triglycerides
HDL-C: High-density lipoprotein cholesterol
LDL-C: Low-density lipoprotein cholesterol
TC: Total cholesterol
FBG: Fasting blood glucose
SUA: Serum uric acid
SCr: Serum creatinine
HR: Hazard ratio
CI: Confidence interval
Q1: Quintile 1
Q2: Quintile 2
Q3: Quintile 3
Q4: Quintile 4
Q5: Quintile 5
